# Impact of HLA Class I Alleles on Timing of HIV Rebound After Antiretroviral Treatment Interruption

**DOI:** 10.20411/pai.v2i3.222

**Published:** 2017-11-27

**Authors:** You Jeong Park, Behzad Etemad, Hayat Ahmed, Vivek Naranbhai, Evgenia Aga, Ronald J. Bosch, John W. Mellors, Daniel R. Kuritzkes, Michael Para, Rajesh T. Gandhi, Mary Carrington, Jonathan Z. Li

**Affiliations:** 1 Brigham and Women's Hospital, Harvard Medical School, Boston, Massachusetts; 2 Harvard College, Cambridge, Massachusetts; 3 Massachusetts General Hospital and Ragon Institute, Harvard Medical School, Boston, Massachusetts; 4 Cancer and Inflammation Program, Laboratory of Experimental Immunology, Leidos Biomedical Research Institute, Frederick National Laboratory for Cancer Research, Frederick, Maryland; 5 Centre for the AIDS Programme of Research in South Africa, Durban, KwaZulu Natal, South Africa; 6 Harvard T.H. Chan School of Public Health, Boston, Massachusetts; 7 University of Pittsburgh, Pittsburgh, Pennsylvania; 8 Ohio State University, Columbus, Ohio

**Keywords:** HLA, HIV reservoir, viral rebound timing, analytic treatment interruption

## Abstract

**Background:**

Identifying host determinants associated with HIV reservoir size and timing of viral rebound after an analytic treatment interruption (ATI) is an important step in the search for an HIV functional cure. We performed a pooled analysis of 103 participants from 4 AIDS Clinical Trials Group ATI studies to identify the association between HLA class I alleles with HIV reservoir size and viral rebound timing.

**Methods:**

Total HIV DNA and cell-associated HIV RNA (CA-RNA) were quantified in pre-ATI peripheral blood mononuclear cell samples, and residual plasma viremia was measured using the single-copy assay. HLA class I typing was performed, and we generated an odds ratio (OR) of predicted HLA effect on HIV viremia control for each individual and compared this with time to viral rebound, and levels of HIV DNA and CA-RNA.

**Results:**

There was no significant association between the HLA ORs and levels of HIV DNA or CA-RNA, but carriage of protective HLA-B alleles (lower OR scores) was associated with delayed viral rebound (*P* = 0.02). Higher OR scores at the HLA-C locus were associated with longer duration of ART treatment (*P* = 0.02) and this trend was also seen with the combined OR score (*P* < 0.01). Individuals with protective HLA-B alleles had delayed viral rebound after treatment interruption that was not explained by differences in baseline reservoir size.

**Conclusions:**

The results indicate the vital role of cellular host immunity in preventing HIV rebound and the importance of taking into account the HLA status of study participants being evaluated in trials for an HIV cure.

## INTRODUCTION

Although antiretroviral therapy (ART) is effective at suppressing HIV replication in infected individuals, the virus persists in a stable pool of resting CD4^+^ T cells in the form of latent provirus that almost inevitably rebounds when treatment is stopped [1]. Eradication of HIV-1 disease necessitates the elimination of this latent reservoir, and identifying host determinants associated with latency and reservoir size in patients receiving ART is an important step in the search for an intervention that provides sustained, long-term ART-free virological remission [2].

One of the main host genetic determinants that influences the control of HIV and disease progression is the human leukocyte antigen (HLA) class I locus [3]. Specific HLA class I alleles like HLA-B*57 and B*27 have consistently been shown to display protective effects and are associated with viral load control and delayed progression to AIDS [4, 5], while the allelic group HLAB*35Px is associated with accelerated disease progression [6]. A combined analysis of 9 HIV cohorts has since provided additional details for HLA class I allele-specific effects in maintaining HIV control [7]. Using the results from that study, we examined the association between alleles at 3 HLA class I loci (HLA-A, B, and C) and both reservoir size and viral dynamics after treatment interruption in participants of the AIDS Clinical Trials Group (ACTG) who underwent an analytic treatment interruption (ATI). Identifying host genetic factors influencing the size of the infected cell population will provide insights into the mechanisms behind viral control after treatment interruption with implications for the efforts to induce ART-free HIV remission.

## METHODS

### Study Population

Participants from 4 ACTGATI studies (A5024 [8], A5068 [9], A5170 [10], and A5197 [11]) were included if they were receiving suppressive ART, received no immunological interventions (eg, therapeutic vaccination), had HIV-1 RNA less than 50 copies/mL at the time of ATI, and had samples available for HLA typing. These participants received ART during chronic infection and have been previously described in an analysis showing the virological predictors of HIV rebound timing [12]. Written informed consent was provided by all study participants for use of stored samples in HIV-related research. This study was approved by the Partners Institutional Review Board.

### Human leukocyte antigen typing

HLA class I typing was performed following the PCR-SSOP (sequence-specific oligonucleotide probing) and the PCR-SBT (sequence based typing) protocols recommended by the 13th International Histocompatibility Workshop (http://www.ihwg.org). Each HLA allele was assigned an odds ratio (OR) for its effects on HIV disease progression based on a previously published multivariable logistic regression analysis of HIV-1 controllers and non-controllers (Supplementary Table 4) [7]. Controllers refer to HIV-1 infected individuals whose mean viral loads are maintained at less than 2,000 copies/mL of plasma despite not receiving ART. Non-controllers refer to individuals whose mean viral loads surpass 10,000 copies/mL. Based on this model, alleles with OR values less than 1 are associated with the protective phenotype seen in controllers while OR values greater than 1 are associated with the unfavorable phenotype of rapid disease progression. For our analysis, alleles that did not reach significance in the model were given an OR value of 1 and defined as neutral. For each participant, the OR's of the 2 alleles in each HLA locus were multiplied to calculate OR_A_, OR_B_, and OR_C_ for the HLA-A, B, and C locus, respectively. The OR scores for all loci were also multiplied to produce a cumulative OR score (OR_ABC_) to reflect the combined effects of all HLA alleles. All HLA B*35Px group alleles (B*3502, 3503, 3504, and B*5301) [6] were assigned the same OR values as B*3502.

As a sensitivity analysis, participants were also grouped into 3 discrete categories: protective, neutral, or unfavorable groups. Participants with at least 1 protective and no unfavorable alleles were categorized as having “protective” HLA genotypes, those with at least 1 unfavorable allele and no protective alleles were categorized as “unfavorable”, and those with 1 protective and 1 unfavorable allele or those with neither protective nor unfavorable alleles were categorized as “neutral”. Categorization of alleles was based on the previously described OR values [7]. HLA-B*57/27 alleles were considered protective regardless of the last 2 digits, and HLA-B*35Px were considered unfavorable for all races.

### HIV-1 reservoir quantification

Cell-associated HIV-1 RNA (CA-RNA) and DNA were isolated from cryopreserved PBMCs using the AllPrep DNA/RNA Mini Kit (Qiagen). Cellular integrity for RNA analysis was assessed by the measurement of total extracted RNA and evaluation of the IPO-8 housekeeping gene [13]. Unspliced CA-RNA and total HIV DNA were quantified using a real-time PCR approach with primers/probes targeting conserved regions of HIV LTR/gag as previously described [14].

### CD4^+^ cell count and pre- and post-ATI viral load measurements

The CD4^+^ cell count before ATI and plasma HIV-1 RNA were defined as the most recent measurements on or before the date of ART discontinuation. The timing of viral rebound was defined as a confirmed viral load ≥ 200 copies/mL or (2) a single viral load ≥1000 copies/mL. Viral load set point was defined as the mean viral load between weeks 12 and 16. The timing of viral rebound was categorized as early (≤ 4 weeks), intermediate (5 to 8 weeks), or delayed (> 8 weeks) after ATI.

## STATISTICAL ANALYSIS

Associations between OR scores (OR_A_, OR_B_, OR_C_, and OR_ABC_) and reservoir size measurements or viral rebound times were analyzed using Spearman correlation, Kruskal-Wallis, Wilcoxon rank sum, and Fisher's exact tests. Associations between categorical HLA-groups and reservoir size measurements or viral rebound times were also analyzed using Fisher's exact tests.

## RESULTS

### Study participants and baseline characteristics

A total of 103 participants were included from the pooled ACTG studies with ATI. Table 1 lists their baseline characteristics. Participants in this study had a median age of 42 years, 90% were male, and 83% were white. The median (Q1, Q3) CD4^+^ cell count at the start of ATI was 843 (687, 1042) cells/μL. Participants had received ART for a median of 5.3 years.

**Table 1.** Baseline characteristics of participants included in the study.

Characteristic	Total (N=103)
Sex, male, n (%)	93 (90%)
Age, median years (Q1, Q3)	42 (38, 50)
Race/Ethnicity, n (%)	
White	85 (83%)
Black	18 (17%)
Nadir CD4^+^ cell count, median cells/μL (Q1, Q3)	422 (351, 544)
Pre-ATI CD4^+^ cell count, median cells/μL (Q1, Q3)	850 (686, 1048)
Duration of ART, median years (Q1, Q3)	5.3 (3.2, 6.6)
NNRTI-based ART	65 (63%)
Source study, n (%)	
A5024	7 (7%)
A5068	12 (12%)
A5170	62 (60%)
A5197	22 (21%)

NNRTI, non-nucleoside reverse transcriptase inhibitor; ART, antiretroviral therapy

**Figure 1.**
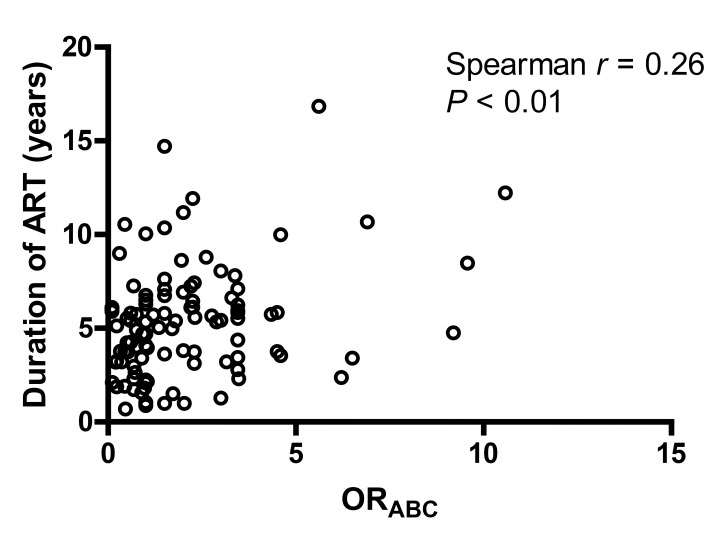
**Correlation between OR_ABC_ and duration of ART.** OR_ABC_ represents an overall assessment of protective status and is calculated by multiplying the ORs of HIV control of all 6 alleles at the A, B, and C loci. A lower OR_ABC_ is associated with greater control of HIV viremia.

There were no significant associations between background characteristics and the estimated effect of each HLA locus individually (OR_A_, OR_B_ or OR_c_) and the overall effect (HLA_ABC_) scores, with the exception of ART duration (Supplementary Tables 1–3). A positive correlation was detected between OR_ABC_ and the duration of ART (Spearman *r* = 0.26, *P* < 0.01, Figure 1), indicating that participants harboring HLA alleles associated with greater risk of HIV non-control had been treated with ART for a longer period of time. A similar trend could be seen for each of the individual HLA loci, but only the ORc analysis reached statistical significance (Spearman *r* = 0.24, *P* = 0.01). These results were consistent with the categorical analysis where protective HLA-C alleles were associated with a shorter duration of ART (protective vs neutral: median 2.5 vs 5.3 years, exact Wilcoxon *P* < 0.01; protective vs unfavorable: median 2.5 vs 5.8 years, Wilcoxon *P* < 0.01).

### Protective effect of HLA is associated with delayed viral rebound timing

**Figure 2.**
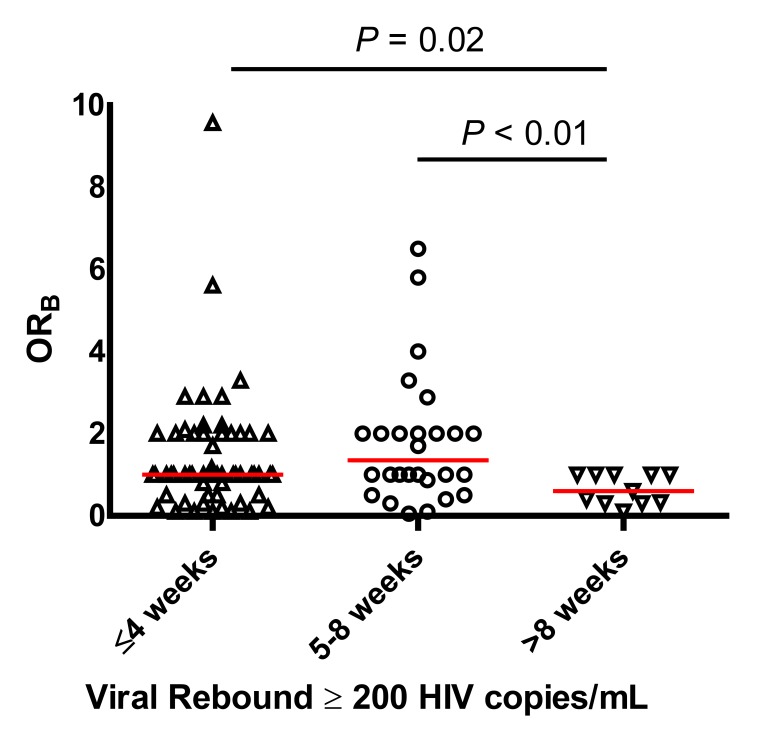
**Association between OR_B_ and timing of viral rebound.** OR_B_ represents the protective status at the HLA-B locus and is calculated for each individual by multiplying the OR estimate of HIV non-control for each of the 2 alleles carried by that individual.

**Supplementary Figure 1.**
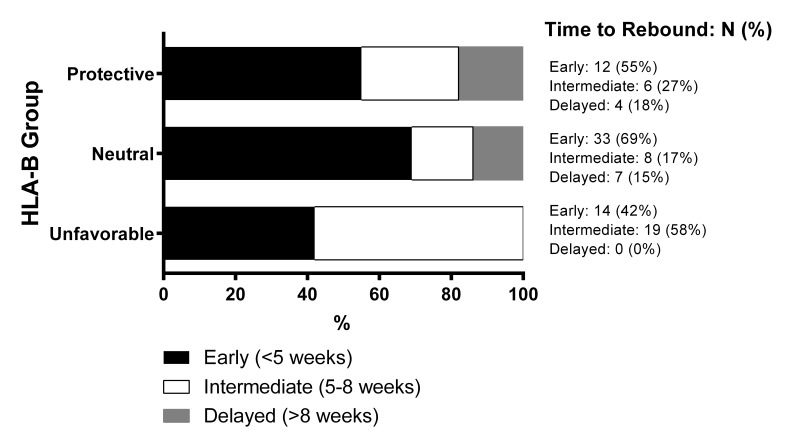
**Univariate analysis of associations at HLA-B locus.** Proportions of participants in HLA-B groups grouped based on their time to viral rebound to ≥ 200 HIV RNA copies/mL.

Participants with delayed viral rebound were found to have significantly more protective HLA-B alleles (lower OR_B_ scores) compared to those with early or intermediate rebound times (early vs delayed: median OR_B_ 1 vs 0.6, *P* = 0.02; intermediate vs delayed: median 1.35 vs 0.6, Figure 2). These results were consistent with the categorical analysis, which showed that there were signifi-cant differences in the distribution of viral rebound timing by HLA-B category (*P* = 0.02, Supplementary Figure 1). The results were largely unchanged with the single ≥ 1,000 HIV-1 RNA copies/mL definition of viral rebound. No significant associations were found in the protective effect of the HLA alleles and either pre-ATI levels of HIV DNA, CA-RNA, or the post-ATI viral load set point.

## DISCUSSION

In this pooled analysis of ACTG studies with ATI, we evaluated the association between HLA class I alleles with HIV reservoir size and post-ATI viral dynamics using a new method of assessing the additive effect of alleles at multiple loci. We found that the presence of protective HLA-B alleles was associated with delayed viral rebound after treatment interruption that was not readily explained by differences in baseline reservoir size. One key immune response associated with control of viral replication is the HIV-1-specific CD8^+^ T lymphocyte responses which are modulated by the host's HLA class I molecules [15]. The role that virus-specific T lymphocytes may play in the control of viral replication is suggested by the association between the appearance of HIV-1-specific CD8^+^ T lymphocytes in peripheral blood with initial decline in viremia during acute infection [16]. This link is further supported by the loss of the ability to control viral replication in macaques infected with simian immunodeficiency virus (SIV) once depleted of CD8^+^ lymphocytes [17]. Although the complete mechanism underlying HLA modulation of HIV disease progression is not well understood, class I alleles associated with the protective phenotype of slower disease progression strongly contribute to the total HIV-specific CD8^+^ T lymphocyte response during acute infection [18]. In this study, higher OR scores (ie, more unfavorable HLA alleles) were associated with a longer time receiving ART. One possible explanation is that unfavorable HLA alleles may lead to more rapid disease progression and therefore to patients starting ART earlier.

To our knowledge, this is the first study demonstrating that protective HLA alleles, specifically in the HLA-B loci, are associated with delayed time to viral rebound, indicating the vital role that cellular host immunity has in preventing HIV rebound after treatment interruption. Given the lack of evidence for an association between HLA alleles and pre-ATI reservoir size (HIV DNA) or activity (CA-RNA), this effect may be mediated by the identification and control of reactivated T cells and viral spread after ART discontinuation. The results also suggest that boosting T-cell-mediated immune responses could be an important component of efforts to induce sustained ART-free HIV remission. In the SPARTAC study, participants with unfavorable HLA alleles had higher HIV DNA levels prior to ART initiation, and only pre-ART levels of T-cell exhaustion predicted the time of viral rebound [19]. However, the SPARTAC study only evaluated participants who initiated ART during early HIV infection, and their sample size was less than half that of this study, limiting the authors' ability to assess HLA-mediated effects. Despite the fact that protective class I HLA alleles are associated with the spontaneous control of HIV, these alleles were not found to be enriched in the VISCONTI cohort of post-treatment controllers [20]. One potential explanation is that while the VISCONTI study limited the categorization of protective HLA alleles to HLA-B*27 and B*57, our analysis provided a more comprehensive and nuanced approach to assessing HLA-related effects. It is also possible that non-HLA-mediated immune responses are needed for sustained HIV remission. In summary, individuals with protective HLA-B alleles had delayed viral rebound after treatment interruption that was not explained by differences in baseline reservoir size. The results indicate the vital role of cellular host immunity in preventing HIV rebound and the importance of taking into account the HLA status of study participants being evaluated in cure trials for HIV because an imbalance in HLA types between study arms may have an unexpected effect on the outcome.

## Figures and Tables

**Supplementary Table 1.** Baseline characteristics for HLA-A groups

Characteristic	HLA-A		
	Protective (n = 7)	Neutral (n = 39)	Unfavorable (n=57)
Sex, male, n (%)	7 (100%)	34 (87%)	51 (89%)
Age, median years (Q1, Q3)	38 (33, 60)	42 (36, 48)	43 (39, 50)
Race/Ethnicity, n (%)			
White	7 (100%)	24 (62%)	54 (95%)
Black	0 (0%)	15 (38%)	3 (5%)
Nadir CD4^+^ cell count, median cells/μL (Q1, Q3)	476 (402, 587)	396 (305, 505)	422 (350, 566)
Pre-ATI CD4^+^ cell count, median cells/μL (Q1, Q3)	905 (798, 1161)	863 (617, 1099)	832 (681, 1010)
Pre-ART viral load, median log_10_ HIV RNA copies/mL (Q1, Q3)	4.0 (3.3, 4.7)	4.4 (3.8, 4.9)	4.4 (4.0, 4.9)
CA-DNA, median log_10_ copies/10^6^ PBMCs (Q1, Q3)	2.3 (2.2, 2.9)	2.4 (1.9, 3.1)	2.3 (1.7, 2.9)
CA-RNA, median log_10_ copies/10^6^ PBMCs (Q1, Q3)	2.0 (1.1, 2.6)	1.7 (1.1, 2.3)	1.8 (1.1, 2.3)
Duration of ART, median years (Q1, Q3)	3.8 (2.6, 5.3)	5.4 (3.2, 6.5)	5.4 (3.3, 7.2)
NNRTI-based ART	4 (57%)	19 (49%)	41 (72%)
Source study, n (%)			
A5024	0 (0%)	3 (8%)	4 (7%)
A5068	0 (0%)	8 (21%)	4 (7s%)
A5170	5 (71%)	22 (56%)	35 (61%)
A5197	2 (29%)	6 (15%)	14 (25%)

**Supplementary Table 2.** Baseline characteristics for HLA-B groups

Characteristic	HLA-B		
	Protective (n = 22)	Neutral (n = 48)	Unfavorable (n = 33)
Sex, male, n (%)	19 (86%)	41 (85%)	32 (97%)
Age, median years (Q1, Q3)	42 (38, 46)	43 (38, 49)	41 (48, 53)
Race/Ethnicity, n (%)			
White	18 (82%)	43 (90%)	24 (73%)
Black	4 (18%)	5 (10%)	9 (27%)
Nadir CD4^+^ cell count, median cells/μL (Q1, Q3)	443 (383, 536)	413 (353, 544)	449 (328, 568)
Pre-ATI CD4^+^ cell count, median cells/μL (Q1, Q3)	876 (626, 1030)	859 (688, 1061)	800 (687, 1127)
Pre-ART viral load, median log_10_ HIV RNA copies/mL (Q1, Q3)	4.2 (3.9, 4.6)	4.3 (3.7, 4.9)	4.5 (3.8, 5.0)
CA-DNA, median log_10_ copies/10^6^ PBMCs (Q1, Q3)	2.1 (1.7, 2.8)	2.4 (1.9, 2.9)	2.4 (1.7, 3.1)
CA-RNA, median log_10_ copies/10^6^ PBMCs (Q1, Q3)	1.6 (1.1, 2.1)	1.8 (1.1, 2.3)	2.0 (1.1, 2.5)
Duration of ART, median years (Q1, Q3)	4.2 (2.8, 5.8)	5.4 (3.3, 6.7)	5.4 (3.3, 8.3)
NNRTI-based ART	12 (55%)	34 (71%)	18 (55%)
Source study, n (%)			
A5024	3 (14%)	3 (6%)	1 (3%)
A5068	0 (0%)	7 (15%)	5 (15%)
A5170	14 (64%)	29 (60%)	19 (58%)
A5197	5 (23%)	9 (19%)	8 (24%)

**Supplementary Table 3.** Baseline characteristics for HLA-C groups

Characteristic	HLA-C		
	Protective (n = 8)	Neutral (n = 79)	Unfavorable (n = 16)
Sex, male, n (%)	7 (88%)	71 (90%)	14 (88%)
Age, median years (Q1, Q3)	38 (35, 51)	43 (38, 50)	43 (40, 52)
Race/Ethnicity, n (%)			
White	5 (63%)	64 (81%)	16 (100%)
Black	3 (37%)	15 (19%)	0 (0%)
Nadir CD4^+^ cell count, median cells/μL (Q1, Q3)	451 (402, 510)	413 (325, 582)	422 (363, 524)
Pre-ATI CD4^+^ cell count, median cells/μL (Q1, Q3)	799 (601, 1310)	852 (653, 1023)	852 (764, 1186)
Pre-ART viral load, median log_10_ HIV RNA copies/mL (Q1, Q3)	4.5 (3.8, 5.3)	4.3 (3.8, 4.7)	4.9 (4.0, 5.5)
CA-DNA, median log_10_ copies/10^6^ PBMCs (Q1, Q3)	2.7 (1.7, 3.3)	2.3 (1.8, 3.1)	2.5 (1.7, 2.7)
CA-RNA, median log_10_ copies/10^6^ PBMCs (Q1, Q3)	1.8 (1.2, 2.6)	1.8 (1.1, 2.3)	1.8 (1.1, 2.3)
Duration of ART, median years (Q1, Q3)	2.5 (1.6, 4.3)	5.3 (3.4, 6.7)	5.8 (3.5, 7.6)
NNRTI-based ART	5 (63%)	50 (63%)	9 (56%)
Source study, n (%)			
A5024	1 (13%)	6 (8%)	0 (0%)
A5068	0 (0%)	9 (11%)	3 (19%)
A5170	6 (74%)	46 (58%)	10 (63%)
A5197	1 (13%)	18 (23%)	3 (19%)

**Supplementary Table 4.** Effect of individual class I alleles on viral control (controllers vs non-controllers) based on a logistic regression model with stepwise selection including all HLA class I alleles with phenotypic frequencies of >2% (Table S5 in [7]).

White				Black			
Allele	*P*	OR	95% CI	Allele	*P*	OR	95% CI
A*01:01	2E-11	2.3	1.8-2.9	A*23:01	3E-04	1.9	1.3-2.7
A*02:01	4E-05	1.5	1.2-1.8	A*36:01	4E-04	4.3	1.9-9.8
A*25:01	1E-03	0.5	0.3-0.8	A*03:01	4E-02	0.7	0.5-1.0
A*31:01	5E-03	0.6	0.4-0.9	B*57:03	2E-22	0.1	0.1-0.2
A*68:02	3E-02	0.5	0.3-0.9	B*45:01	8E-05	3.3	1.8-5.9
B*57:01	3E-42	0.1	0.1-0.2	B*35:01	3E-04	2.2	1.4-3.3
B*27:05	3E-13	0.3	0.2-0.4	B*15:10	1E-03	2.6	1.5-4.7
B*07:02	2E-07	2.0	1.5-2.6	B*81:01	2E-03	0.4	0.2-0.7
B*52:01	4E-06	0.3	0.2-0.5	B*58:02	3E-03	2.5	1.4-4.5
B*14:02	2E-05	0.5	0.3-0.7	B*18:01	5E-03	2.5	1.3-4.8
B*13:02	3E-05	0.4	0.3-0.6	B*07:02	1E-02	1.7	1.1-2.5
B*40:01	4E-04	2.0	1.4-2.9	B*14:02	4E-02	0.6	0.3-1.0
B*38:01	5E-04	2.9	1.6-5.2	C*12:03	8E-05	0.3	0.2-0.6
B*18:01	7E-04	2.0	1.3-3.0	C*08:04	4E-03	0.3	0.1-0.7
B*40:02	4E-03	0.5	0.3-0.8	C*05:01	2E-02	0.5	0.3-0.9
B*58:01	2E-02	0.5	0.3-0.9				
B*35:02	3E-02	2.9	1.1-7.9				
B*55:01	4E-02	2.1	1.0-4.4				
C*14:02	1E-04	0.4	0.2-0.6				
C*04:01	1E-03	1.5	1.2-2.0
